# Complete Genome Sequence of *Clostridium septicum* Strain CSUR P1044, Isolated from the Human Gut Microbiota

**DOI:** 10.1128/genomeA.00922-16

**Published:** 2016-09-08

**Authors:** Samia Benamar, Nadim Cassir, Aurélia Caputo, Frédéric Cadoret, Bernard La Scola

**Affiliations:** Unité de Recherche sur les Maladies Infectieuses et Tropicales Emergentes, CNRS, UMR 6236, IRD 198, Faculté de médecine, Marseille Cedex, France

## Abstract

*Clostridium septicum* is one of the first pathogenic anaerobes to be identified. Here, we announce the genome draft sequence of *C. septicum* strain CSUR P1044 isolated from the gut of a healthy adult. Its chromosome genome consists of 3.2 Mbp with a plasmid of 32 Kbp. *C. septicum* strain CSUR P1044 has a G+C content of 27.5%, and is composed of 3,125 protein-coding genes together with 103 RNA genes, including 22 rRNA genes.

## GENOME ANNOUNCEMENT

*Clostridium septicum*, a Gram-positive, spore-forming bacillus, is the first anaerobic opportunistic bacterium ever cultivated, discovered by Pasteur in 1877 (1). Since then, it has been reported to be present in the environment and in the gut microbiota of humans and animals (2). *C. septicum* is now recognized as a causative agent of both traumatic and nontraumatic gas gangrene, but cases of arthritis and aortitis have also been described (2–4). Infection primarily occurs in adults with severe hematological malignancies and colorectal cancer, as well as in children with severe neutropenia (5, 6). The major virulence factor produced by *C. septicum* is a pore-forming alpha-toxin encoded by the *csa* gene, which appears to be carried by all strains of this bacterium (7).

We report the complete genome sequence of *C. septicum* strain CSUR P1044 isolated from the gut of a healthy adult.

The genomic DNA was sequenced using a combination of paired-end libraries (average insert of 250 bp) on an Illumina MiSeq. The generated reads were assembled using the SPAdes genome assembler (8). The assembled genome counts 79 contigs regrouped into 80 scaffolds. The estimated genome size was 3,266,706 bp with a G+C content of 27.5%. A single contig was identified as a plasmid, with 32,264 bp sequence size and 28% G+C content value. The average read depth of the *C. septicum* plasmid (84.20×) compared to the chromosome (23.00×) implies that the plasmid is present as approximately three to four copies per cell.

The draft genome sequence of *Clostridium septicum* is smaller than those of *Clostridium sartagoforme* AAU1*, Clostridium disporicum, Clostridium beijerinckii* AAU1*, Clostridium paraputrificum, Clostridium colicanis* (3.98, 3.80, 6.96, 3.56, and 3.51 Mb, respectively), but larger than those of *Clostridium chauvoei* JF4335 (2.81 Mb). The G+C content value of *Clostridium septicum* is smaller than those of *Clostridium chauvoei* JF4335, *Clostridium sartagoforme* AAU1, *Clostridium disporicum*, *Clostridium beijerinckii* AAU1, *Clostridium paraputrificum* (27.6, 27.9, 27.9, 30.5, and 29.6%, respectively), but higher than those of *Clostridium colicanis* (26.1%).

The genome is predicted to contain 3,125 protein-coding sequences (CDS), 22 rRNA operons, and 81 tRNAs using RAST (Rapid Annotation using Subsystem Technology) (9). The genome was visualized using Artemis software (10). The plasmid was predicted to harbor 54 protein CDS and shares some features with the plasmid pCS2 in *Clostridium sordellii*, such as a phage terminase (Genbank accession number CEO20805).

We identified the *csa* gene, located in the chromosome. It encodes to an alpha-toxin that has structural similarity to aerolysin from *Aeromonas hydrophilia* (11). No toxin was identified in the plasmid.

### Accession number(s).

The complete genome sequences of *Clostridium septicum* strain CSUR P1044 have been deposited in GenBank under accession numbers FLTT01000001 to FLTT01000078 (chromosome) and FLTT01000079 (plasmid).
